# 2-Aza­niumyl-4-(ethyl­carbamo­yl)butano­ate: the zwitterionic form of the amino acid theanine

**DOI:** 10.1107/S1600536810050701

**Published:** 2010-12-11

**Authors:** Shou-Kai Kang, Yue-Hu Chen, Shao-Song Qian, Hao Hu

**Affiliations:** a School of Life Science, ShanDong University of Technology, ZiBo 255049, People’s Republic of China

## Abstract

In the title zwitterion, C_7_H_14_N_2_O_3_, the ethyl­amino and the 5-oxo groups are positionally disordered with occupancy ratios of 0.50:0.50 and 0.70:0.30, respectively. The terminal ethyl –CH_3_ group undergoes considerable thermal motion. In the crystal, mol­ecules are linked *via* N—H⋯O hydrogen bonds, forming a two-dimensional arrangement propagating in the *bc* plane.

## Related literature

For details of the physiological activity of the amino acid theanine, commonly found in certain teas, see: Li *et al.* (2006 ▶).
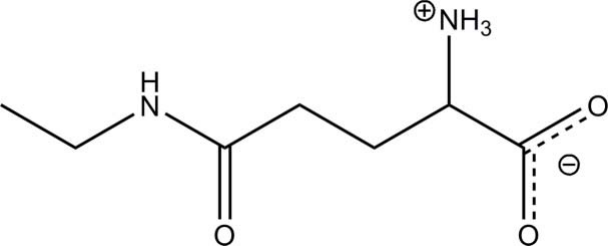


## Experimental

### 

#### Crystal data

C_7_H_14_N_2_O_3_
*M* *_r_* = 174.20
Monoclinic, 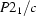

*a* = 19.606 (6) Å
*b* = 4.7904 (15) Å
*c* = 9.812 (3) Å
β = 90.501 (6)°
*V* = 921.5 (5) Å^3^
*Z* = 4
Mo *K*α radiation
μ = 0.10 mm^−1^
*T* = 273 K
0.15 × 0.12 × 0.06 mm

#### Data collection

Bruker SMART CCD area-detector diffractometer
5580 measured reflections
2218 independent reflections
1276 reflections with *I* > 2σ(*I*)
*R*_int_ = 0.039

#### Refinement

*R*[*F* ^2^ > 2σ(*F* ^2^)] = 0.051
*wR*(*F* ^2^) = 0.147
*S* = 1.01
2218 reflections
148 parameters
26 restraints
H-atom parameters constrained
Δρ_max_ = 0.23 e Å^−3^
Δρ_min_ = −0.20 e Å^−3^

### 

Data collection: *SMART* (Bruker, 2007 ▶); cell refinement: *SAINT* (Bruker, 2007 ▶); data reduction: *SAINT*; program(s) used to solve structure: *SHELXS97* (Sheldrick, 2008 ▶); program(s) used to refine structure: *SHELXL97* (Sheldrick, 2008 ▶); molecular graphics: *PLATON* (Spek, 2009 ▶); software used to prepare material for publication: *SHELXTL* (Sheldrick, 2008 ▶).

## Supplementary Material

Crystal structure: contains datablocks global, I. DOI: 10.1107/S1600536810050701/su2225sup1.cif

Structure factors: contains datablocks I. DOI: 10.1107/S1600536810050701/su2225Isup2.hkl

Additional supplementary materials:  crystallographic information; 3D view; checkCIF report

## Figures and Tables

**Table 1:** Hydrogen-bond geometry (Å, °)

*D*—H⋯*A*	*D*—H	H⋯*A*	*D*⋯*A*	*D*—H⋯*A*
N1—H1*A*⋯O2^i^	0.89	1.89	2.776 (2)	174
N1—H1*B*⋯O1^ii^	0.89	1.96	2.8332 (19)	165
N1—H1*C*⋯O1^iii^	0.89	1.97	2.850 (2)	171
N2—H2⋯O3^iv^	0.86	2.16	2.93 (2)	149
N2′—H2′⋯O3′^iv^	0.86	2.01	2.85 (3)	166

Symmetry codes: (i) 
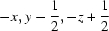
; (ii) 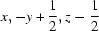; (iii) 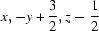; (iv) 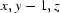.

**Fig. 1.**
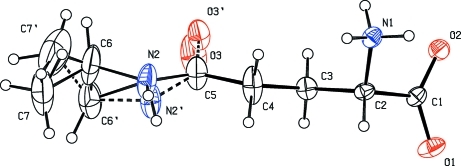
The molecular structure of the title compound, showing the atom-labelling scheme. Displacement ellipsoids are drawn at the 30% probability level. The ethylamino group (atoms N2,C6,C7/N2',C6',C7': occupancies 0.5/0.5) and the 5-oxo (O2/O2': occupancies 0.7/0.3) atom are positionally disordered.

**Fig. 2.**
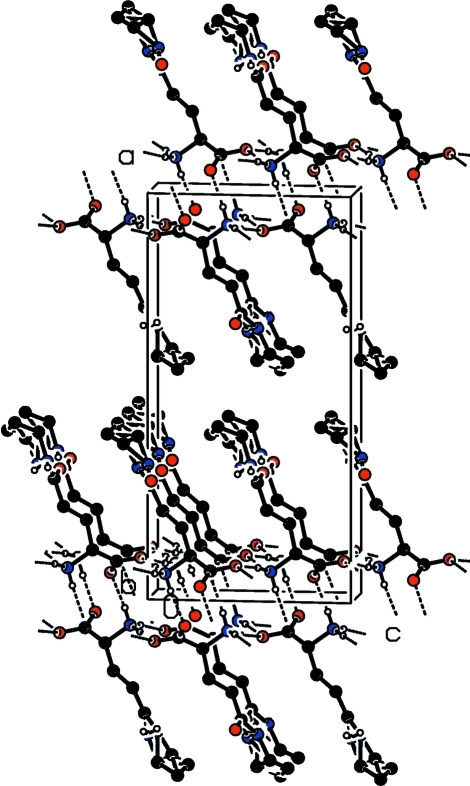
A view along the b-axis of the crystal packing of the title compound. The N-H···O hydrogen bonds are shown as dashed lines - see Table 1 for details (H-atoms not involved in hydrogen bonding have been omitted for clarity).

## supplementary crystallographic information

### Comment

In recent years, increasing attention has been drawn towards the physiological and pharmacological applications of theanine, which besides its favorable taste has been reported to be biologically active promoting relaxation, inhibiting negative effects of caffeine and enhancing anti-tumor activity. Moreover, it has been found to have physiological activities including Neuroprotection and anti-obesity (Li *et al.*, 2006).

The title molecule was found to crystallize in the Zwitter ion form (Fig. 1). The ethylamino group (atoms N2,C6,C7/N2',C6',C7': occupancies 0.5/0.5) and the 5-oxo (O2/O2': occupancies 0.7/0.3) atom are positionally disordered. The terminal ethyl CH_3_ group (C7 and C7') undergoes considerable thermal motion.

In the crystal the molecules are linked via N-H···O hydrogen bonds to form a two-dimensional arrangement propagating in the *bc*-plane (Table 1, Fig. 2).

### Experimental

The title compound was synthesized according to a Chinese Patent (Li, *et al.*, 2006). 20 g of *L*-pyrrolidone carboxylic acid were reacted with 20 g of anhydrous ethylamine in helium gas under a pressure of 7 MPa for 4hr. 23.3 g of the theanine were obtained. The single crystals, of the title compound, suitable for X-ray diffraction analysis, were obtained by the hanging-drop method with water as solvent.

### Refinement

The ethylamino group (atoms N2,C6-C7/N2',C6',C7': occupancies 0.5/0.5) and the 5-oxo (O2/O2': occupancies 0.7/0.3) moiety are positionally disordered. The terminal ethyl CH_3_ group (C7 and C7') undergoes considerable thermal motion. All the H-atoms were placed in geometrical positions and constrained to ride on their parent atoms: N-H = 0.89 and 0.86 Å for NH_3_ and NH H-atoms, respectively, and C—H 0.98, 0.97 and 0.96 Å, for CH, CH_2_ and CH_3_, respectively, with *U*_iso_(H) = k × *U*_eq_(N or C) where k = 1.5 for NH_3_ and CH_3_ H.atoms, and k = 1.2 for all other H-atoms.

### Crystal data

**Table:**

C_7_H_14_N_2_O_3_	*F*(000) = 376
*M**_r_* = 174.20	*D*_x_ = 1.256 Mg m^−^^3^
Monoclinic, *P*2_1_/*c*	Mo *K*α radiation, λ = 0.71073 Å
Hall symbol: -P 2ybc	Cell parameters from 838 reflections
*a* = 19.606 (6) Å	θ = 4.2–23.0°
*b* = 4.7904 (15) Å	µ = 0.10 mm^−^^1^
*c* = 9.812 (3) Å	*T* = 273 K
β = 90.501 (6)°	Prism, colourless
*V* = 921.5 (5) Å^3^	0.15 × 0.12 × 0.06 mm
*Z* = 4	

### Data collection

**Table:**

Bruker SMART CCD area-detector diffractometer	1276 reflections with *I* > 2σ(*I*)
Radiation source: fine-focus sealed tube	*R*_int_ = 0.039
graphite	θ_max_ = 28.3°, θ_min_ = 2.1°
phi and ω scans	*h* = −21→25
5580 measured reflections	*k* = −6→6
2218 independent reflections	*l* = −13→9

### Refinement

**Table:**

Refinement on *F*^2^	Primary atom site location: structure-invariant direct methods
Least-squares matrix: full	Secondary atom site location: difference Fourier map
*R*[*F*^2^ > 2σ(*F*^2^)] = 0.051	Hydrogen site location: inferred from neighbouring sites
*wR*(*F*^2^) = 0.147	H-atom parameters constrained
*S* = 1.01	*w* = 1/[σ^2^(*F*_o_^2^) + (0.0696*P*)^2^] where *P* = (*F*_o_^2^ + 2*F*_c_^2^)/3
2218 reflections	(Δ/σ)_max_ < 0.001
148 parameters	Δρ_max_ = 0.23 e Å^−^^3^
26 restraints	Δρ_min_ = −0.20 e Å^−^^3^

### Special details

**Table:**

Geometry. All esds (except the esd in the dihedral angle between two l.s. planes) are estimated using the full covariance matrix. The cell esds are taken into account individually in the estimation of esds in distances, angles and torsion angles; correlations between esds in cell parameters are only used when they are defined by crystal symmetry. An approximate (isotropic) treatment of cell esds is used for estimating esds involving l.s. planes.
Refinement. Refinement of F^2^ against ALL reflections. The weighted R-factor wR and goodness of fit S are based on F^2^, conventional R-factors R are based on F, with F set to zero for negative F^2^. The threshold expression of F^2^ > 2sigma(F^2^) is used only for calculating R-factors(gt) etc. and is not relevant to the choice of reflections for refinement. R-factors based on F^2^ are statistically about twice as large as those based on F, and R- factors based on ALL data will be even larger.

### Fractional atomic coordinates and isotropic or equivalent isotropic displacement parameters (Å^2^)

**Table:**

	*x*	*y*	*z*	*U*_iso_*/*U*_eq_	Occ. (<1)
O1	0.10121 (7)	0.5612 (3)	0.46220 (12)	0.0493 (4)	
O2	0.05541 (6)	0.8285 (3)	0.30018 (12)	0.0396 (4)	
O3	0.3108 (4)	0.734 (2)	0.0555 (10)	0.099 (3)	0.70
O3'	0.2918 (9)	0.741 (4)	0.013 (2)	0.062 (3)	0.30
N1	0.07561 (7)	0.4367 (3)	0.10890 (14)	0.0357 (4)	
H1A	0.0325	0.4110	0.1338	0.054*	
H1B	0.0881	0.2996	0.0532	0.054*	
H1C	0.0793	0.5997	0.0660	0.054*	
C1	0.08906 (9)	0.6232 (3)	0.34041 (17)	0.0319 (4)	
C2	0.12060 (9)	0.4366 (3)	0.23210 (16)	0.0313 (4)	
H2A	0.1243	0.2458	0.2675	0.038*	
C3	0.19192 (9)	0.5443 (4)	0.19622 (19)	0.0405 (5)	
H3A	0.1879	0.7328	0.1609	0.049*	
H3B	0.2194	0.5525	0.2788	0.049*	
C4	0.22817 (11)	0.3658 (5)	0.0926 (3)	0.0708 (8)	
H4A	0.2382	0.1859	0.1334	0.085*	
H4B	0.1976	0.3338	0.0159	0.085*	
C5	0.29288 (12)	0.4882 (5)	0.0407 (3)	0.0633 (7)	
N2	0.3323 (10)	0.312 (4)	−0.0317 (13)	0.066 (4)	0.50
H2	0.3217	0.1382	−0.0386	0.079*	0.50
C6	0.3928 (6)	0.420 (2)	−0.0976 (13)	0.099 (4)	0.50
H6A	0.4174	0.5415	−0.0355	0.119*	0.50
H6B	0.3798	0.5269	−0.1775	0.119*	0.50
C7	0.4368 (6)	0.183 (3)	−0.1378 (15)	0.137 (5)	0.50
H7A	0.4453	0.0661	−0.0602	0.205*	0.50
H7B	0.4793	0.2540	−0.1713	0.205*	0.50
H7C	0.4145	0.0771	−0.2081	0.205*	0.50
N2'	0.3403 (10)	0.302 (4)	0.0159 (13)	0.069 (4)	0.50
H2'	0.3293	0.1295	0.0275	0.083*	0.50
C6'	0.4104 (5)	0.357 (2)	−0.0298 (10)	0.087 (3)	0.50
H6'1	0.4414	0.2225	0.0113	0.105*	0.50
H6'2	0.4243	0.5420	−0.0011	0.105*	0.50
C7'	0.4137 (8)	0.336 (4)	−0.1820 (13)	0.159 (5)	0.50
H7'1	0.3892	0.1728	−0.2119	0.238*	0.50
H7'2	0.4604	0.3213	−0.2094	0.238*	0.50
H7'3	0.3935	0.4989	−0.2223	0.238*	0.50

### Atomic displacement parameters (Å^2^)

**Table:**

	*U*^11^	*U*^22^	*U*^33^	*U*^12^	*U*^13^	*U*^23^
O1	0.0812 (11)	0.0390 (8)	0.0277 (7)	0.0014 (7)	−0.0001 (6)	0.0010 (6)
O2	0.0481 (8)	0.0319 (7)	0.0389 (7)	0.0056 (6)	0.0051 (6)	0.0004 (6)
O3	0.072 (5)	0.045 (3)	0.181 (8)	−0.013 (3)	0.046 (4)	−0.019 (4)
O3'	0.050 (7)	0.032 (4)	0.104 (7)	−0.001 (5)	0.025 (5)	0.009 (5)
N1	0.0426 (9)	0.0328 (8)	0.0317 (8)	−0.0019 (7)	0.0044 (7)	−0.0035 (6)
C1	0.0394 (10)	0.0263 (9)	0.0301 (9)	−0.0065 (8)	0.0040 (7)	−0.0002 (7)
C2	0.0419 (10)	0.0236 (8)	0.0284 (9)	0.0010 (7)	0.0002 (7)	0.0009 (7)
C3	0.0410 (11)	0.0343 (10)	0.0463 (11)	−0.0018 (8)	0.0019 (8)	−0.0039 (8)
C4	0.0524 (14)	0.0498 (14)	0.111 (2)	−0.0112 (11)	0.0364 (14)	−0.0275 (14)
C5	0.0549 (14)	0.0419 (13)	0.0936 (19)	−0.0060 (11)	0.0271 (13)	−0.0113 (13)
N2	0.068 (7)	0.042 (3)	0.089 (8)	−0.003 (4)	0.045 (6)	−0.013 (5)
C6	0.088 (8)	0.078 (6)	0.134 (9)	0.005 (4)	0.079 (7)	0.008 (6)
C7	0.092 (7)	0.120 (8)	0.199 (13)	0.018 (5)	0.084 (9)	0.003 (7)
N2'	0.048 (4)	0.053 (4)	0.107 (10)	−0.012 (3)	0.032 (7)	−0.010 (7)
C6'	0.059 (5)	0.067 (5)	0.137 (9)	−0.006 (4)	0.038 (5)	−0.001 (5)
C7'	0.125 (12)	0.216 (17)	0.136 (9)	−0.046 (11)	0.055 (8)	0.002 (11)

### Geometric parameters (Å, °)

**Table:**

O1—C1	1.252 (2)	C5—N2	1.351 (18)
O2—C1	1.246 (2)	N2—C6	1.45 (2)
O3—C5	1.237 (10)	N2—H2	0.8600
O3'—C5	1.24 (2)	C6—C7	1.479 (12)
N1—C2	1.490 (2)	C6—H6A	0.9700
N1—H1A	0.8900	C6—H6B	0.9700
N1—H1B	0.8900	C7—H7A	0.9600
N1—H1C	0.8900	C7—H7B	0.9600
C1—C2	1.524 (2)	C7—H7C	0.9600
C2—C3	1.534 (2)	N2'—C6'	1.47 (2)
C2—H2A	0.9800	N2'—H2'	0.8600
C3—C4	1.511 (3)	C6'—C7'	1.499 (13)
C3—H3A	0.9700	C6'—H6'1	0.9700
C3—H3B	0.9700	C6'—H6'2	0.9700
C4—C5	1.491 (3)	C7'—H7'1	0.9600
C4—H4A	0.9700	C7'—H7'2	0.9600
C4—H4B	0.9700	C7'—H7'3	0.9600
C5—N2'	1.31 (2)		
			
C2—N1—H1A	109.5	O3'—C5—N2	120.2 (13)
C2—N1—H1B	109.5	O3—C5—C4	125.2 (5)
H1A—N1—H1B	109.5	O3'—C5—C4	116.5 (9)
C2—N1—H1C	109.5	N2'—C5—C4	113.6 (9)
H1A—N1—H1C	109.5	N2—C5—C4	115.2 (9)
H1B—N1—H1C	109.5	C5—N2—C6	119.1 (15)
O2—C1—O1	125.80 (16)	C5—N2—H2	120.5
O2—C1—C2	117.32 (15)	C6—N2—H2	120.5
O1—C1—C2	116.84 (16)	N2—C6—C7	109.2 (11)
N1—C2—C1	108.93 (14)	N2—C6—H6A	109.8
N1—C2—C3	110.36 (14)	C7—C6—H6A	109.8
C1—C2—C3	109.80 (14)	N2—C6—H6B	109.8
N1—C2—H2A	109.2	C7—C6—H6B	109.8
C1—C2—H2A	109.2	H6A—C6—H6B	108.3
C3—C2—H2A	109.2	C5—N2'—C6'	126.7 (16)
C4—C3—C2	113.51 (15)	C5—N2'—H2'	116.6
C4—C3—H3A	108.9	C6'—N2'—H2'	116.6
C2—C3—H3A	108.9	N2'—C6'—C7'	109.9 (10)
C4—C3—H3B	108.9	N2'—C6'—H6'1	109.7
C2—C3—H3B	108.9	C7'—C6'—H6'1	109.7
H3A—C3—H3B	107.7	N2'—C6'—H6'2	109.7
C5—C4—C3	114.44 (18)	C7'—C6'—H6'2	109.7
C5—C4—H4A	108.7	H6'1—C6'—H6'2	108.2
C3—C4—H4A	108.7	C6'—C7'—H7'1	109.5
C5—C4—H4B	108.7	C6'—C7'—H7'2	109.5
C3—C4—H4B	108.7	H7'1—C7'—H7'2	109.5
H4A—C4—H4B	107.6	C6'—C7'—H7'3	109.5
O3—C5—N2'	117.9 (10)	H7'1—C7'—H7'3	109.5
O3'—C5—N2'	129.3 (13)	H7'2—C7'—H7'3	109.5
O3—C5—N2	119.6 (10)		
			
O2—C1—C2—N1	−31.8 (2)	O3—C5—N2—C6	−3.8 (16)
O1—C1—C2—N1	150.42 (15)	O3'—C5—N2—C6	26.3 (18)
O2—C1—C2—C3	89.14 (19)	N2'—C5—N2—C6	−95 (5)
O1—C1—C2—C3	−88.62 (18)	C4—C5—N2—C6	173.9 (10)
N1—C2—C3—C4	−61.8 (2)	C5—N2—C6—C7	164.9 (13)
C1—C2—C3—C4	178.08 (18)	O3—C5—N2'—C6'	−16.3 (16)
C2—C3—C4—C5	171.3 (2)	O3'—C5—N2'—C6'	12 (2)
C3—C4—C5—O3	−14.3 (6)	N2—C5—N2'—C6'	84 (5)
C3—C4—C5—O3'	−43.0 (10)	C4—C5—N2'—C6'	−177.0 (10)
C3—C4—C5—N2'	144.8 (7)	C5—N2'—C6'—C7'	−94.9 (15)
C3—C4—C5—N2	168.1 (7)		

### Hydrogen-bond geometry (Å, °)

**Table:**

*D*—H···*A*	*D*—H	H···*A*	*D*···*A*	*D*—H···*A*
N1—H1A···O2^i^	0.89	1.89	2.776 (2)	174
N1—H1B···O1^ii^	0.89	1.96	2.8332 (19)	165
N1—H1C···O1^iii^	0.89	1.97	2.850 (2)	171
N2—H2···O3^iv^	0.86	2.16	2.93 (2)	149
N2'—H2'···O3'^iv^	0.86	2.01	2.85 (3)	166

Symmetry codes: (i) −*x*, *y*−1/2, −*z*+1/2; (ii) *x*, −*y*+1/2, *z*−1/2; (iii) *x*, −*y*+3/2, *z*−1/2; (iv) *x*, *y*−1, *z*.
